# Cellular mechanisms regulating synthetic sex ratio distortion in the *Anopheles gambiae* germline

**DOI:** 10.1080/20477724.2020.1803628

**Published:** 2020-10-12

**Authors:** Roya Elaine Haghighat-Khah, Atashi Sharma, Mariana Reis Wunderlich, Giulia Morselli, Louise Anna Marston, Christopher Bamikole, Ann Hall, Nace Kranjc, Chrysanthi Taxiarchi, Igor Sharakhov, Roberto Galizi

**Affiliations:** a Department of Life Sciences, Imperial College London, London, UK; b Department of Entomology, Virginia Polytechnic Institute and State University, Blacksburg, VA, USA; c Department of Cytology and Genetics, Tomsk State University, Tomsk, Russian Federation; d Centre for Applied Entomology and Parasitology, School of Life Sciences, Keele University, UK

**Keywords:** Sex ratio distortion, genetic control, mosquito, malaria, genetics, meiotic checkpoints, meiosis, gene drive, Y-drive, rDNA, end-joining repair, molecular mechanisms, Fluorescence *in situ* hybridization (FISH); sex chromosomes; male-bias; transgenic.

## Abstract

Genetic control strategies aimed to bias the sex of progenies towards males present a promising new paradigm to eliminate malaria-transmitting mosquitoes. A synthetic sex-ratio distortion (SD) system was successfully engineered in *Anopheles gambiae* by exploiting the meiotic activity of the I-PpoI endonuclease targeting ribosomal DNA (rDNA) repeats, exclusively located on the X chromosome. Males carrying the SD construct produce highly male-biased progenies without evident reduction in fertility. In this study, we investigated the fate of X and Y chromosomes in these SD males and found that ratios of mature X:Y-bearing sperm were comparable to wild-type insects, indicating absence of selection mechanisms during sperm maturation. We therefore tested the effect of meiotic cleavage of both X and Y chromosomes in a lab-generated SD strain carrying rDNA on both sex chromosomes, showing fertility comparable to wild-type and a reduced male-bias compared to SD males in which only the X is targeted. Exposure of Y-linked rDNA to I-PpoI cleavage for consecutive generations rapidly restored the male-bias to typical high frequencies, indicating a correlation between the number of cleavable targets in each sex chromosome and the sex-ratios found in the progeny. Altogether our results indicate that meiotic cleavage of rDNA repeats, located in the sex chromosomes of *A. gambiae* SD males, affects the competitiveness of mature sperm to fertilize the female oocyte, thereby generating sex-biased progenies. We also show that the presence of rDNA copies on the Y chromosome does not impair the effectiveness of engineered synthetic SD systems for the control of human malaria mosquitoes.

## Introduction

Malaria is one of the most severe global health problems. Caused by the *Plasmodium* parasite, malaria was responsible for an estimated 435,000 deaths in 2017, mostly in Africa [1]. The *Anopheles gambiae* complex includes the most efficient vectors of human malaria in sub-Saharan Africa. Only the female mosquitoes take human blood meals to obtain essential nutrients for the development of their eggs and transmit the malaria-causing parasites during an infective bite. The use of genetic control to reduce the population of malaria vectors offers a new promising tool to complement existing mosquito control strategies that aim to reduce the public health burden of malaria. One of the most powerful genetic control approaches proposed, known as sex ratio distortion (SD), requires the development of fully fertile *Anopheles* male mosquitoes that are able to produce a normal number of progenies but mostly consisting of males. Their release in wild populations would cause a progressive reduction in the number of malaria-transmitting females and, at the same time, diminish their population size. Naturally occurring male-biased SD systems (also defined as natural ‘meiotic drivers’), found in *Aedes aegypti* [2] and *Culex pipiens* [3] males, are associated with preferential breakage of sex chromosomes during the first meiotic division [2,3].

Like in humans, *Anopheles* males are the heterogametic sex carrying both heteromorphic sex chromosomes (XY), whilst females are homogametic (XX). Thus, the genetic engineering of such synthetic sex distorters can be achieved by selectively disrupting the viability of the male gametes carrying the X chromosomes making only Y-bearing sperm available to fertilize the wild-type females’ oocytes [4,5]. One way to achieve this goal is to use endonucleases to target repetitive sequences uniquely present within the X chromosome during male meiosis (the process driving the formation of the haploid gametes). This strategy is generally identified as ‘X-shredding’.

Suitable for this purpose, the rDNA is clustered in hundreds of copies that are exclusively localised on the X chromosome of the main human malaria vectors, including the extensively studied *A. gambiae* G3 and *A. arabiensis* Dongola lab strains [6]. It is also known that some species of the *A. gambiae* complex, such as *A. merus, A. quadriannulatus* and the *A. gambiae* ASEMBO lab strain (also referred to as Asembo Bay or AB), contain an rDNA locus on both X and Y sex chromosomes [7,8]. These findings have raised uncertainties about the effectiveness of SD control strategies via X-shredding in the vector species where the targeted rDNA repeats may not be exclusively linked to the X chromosome.

A synthetic SD or ‘X-shredder’ was engineered in the *A. gambiae* G3 lab colony by expressing, under the transcriptional control of the *β2-tubulin* promoter (β2-tub), variants of the I-PpoI endonuclease with reduced stability and targeting a ~ 15 bp DNA sequence uniquely present within the highly conserved 28S rDNA subunit [9]. The β2-tub promoter is specifically active in the testes during male meiosis and is thought to drive transcription as early as the first meiotic division [10]. Males carrying single insertions of the β2-tub::I-*Ppo*I transgene at various locations produced mostly males as a consequence of X-shredding at the rDNA cluster. The ^gfp^124L-2 transgenic line examined in this study, referred to herein as Ag(PMB)1, carries the autosomal β2-tub::I-PpoI transgene and produces approximately 95% male progeny without detectable effects in fertility and fecundity [9]. Similarly, the X-linked rDNA in the *A. gambiae* G3 lab strain was targeted by a β2-tub driven Cas9 endonuclease, producing highly male-biased progenies combined with full fertility [11].

Sustained mass releases of the autosomal I-*Ppo*I or CRISPR-Cas9 SD systems could contribute toward local suppression of female mosquitoes at a village-scale [12]. Moreover, these systems have great potential for area-wide suppression at a country or continental scale if combined with a ‘gene-drive’ mechanism to bias its own inheritance at a greater than mendelian rate. For example if the X-targeting SD endonuclease is inserted into a meiotically active region of the Y chromosome, also referred as ‘Y-drive’, a copy of the SD gene will be inherited by all surviving male offspring [4,5–13]. However, the same β2-tub::I-*Ppo*I transgene, highly active when inserted at autosomal loci, is silenced when inserted on the *A. gambiae* Y chromosome [14]. This is due to a process known as meiotic sex chromosome inactivation (MSCI) [15], acting from the meiotic prophase I until completion of spermiogenesis [16], which complicates the engineering of an active ‘Y-drive’ in *A. gambiae*. Alternatively, the SD can be combined with a homing CRISPR-based nuclease system to bias its inheritance [17–21], as recently shown in *A. gambiae* [21].

To date, the cellular mechanisms following selective breakage and selection of sex chromosomes, which results in male-biased progeny, remains elusive for both naturally occurring and synthetic mosquito SD systems. In a previous study, *Ae. aegypti* males carrying the naturally occurring SD produced 29% fewer spermatozoa compared to the wild-type males of a reciprocal cross, indicating a possible meiotic checkpoint responsible for detecting and removing damaged X-sperm, though not by enough to explain the resulting male bias with just 3.8% of females being generated [2]. Synthetic *A. gambiae* SD males, such as Ag(PMB)1, produce highly male biased progenies without a reduction in the total number of individuals generated [9,11].

The aim of this study was to investigate the two plausible mechanisms that may determine the generation of extreme male bias in the *A. gambiae* SD Ag(PMB)1 transgenic line: (1) selective removal of haploid germline cells carrying the ‘shredded’ X chromosome, possibly regulated by meiotic checkpoints triggered by the selective breakage of the X chromosome; or (2) pre- or postcopulatory sperm competition between damaged X-bearing sperm and unaffected Y-bearing sperm.

To test these hypotheses, we visualized the sex chromosomes in spermatozoa using fluorescence *in situ* hybridization (FISH) to count the proportion of X and Y-bearing sperm at pre and postcopulatory stages. We also introduced the Y chromosome of the *A. gambiae* ASEMBO strain (Y^AS^) into the Ag(PMB)1 genetic background to assess the phenotypic consequences resulting from the simultaneous cleavage of both X and Y chromosomes during male meiosis.

## Results and discussion

### Meiotic expression of I-PpoI does not imbalance the ratio of X and Y bearing spermatozoa transferred to females by Ag(PMB)1 males

To investigate whether sperm carrying the shredded X chromosome were removed before reaching maturation, fluorescence *in situ* hybridization (FISH), using X and Y-specific probes, was performed on dissected Ag(PMB)1 testes as well as the spermathecae of females mated with Ag(PMB)1 males. The ratio of X and Y-bearing sperm was compared to the ratio of males and females found in their progeny. Confocal analysis showed that there was no significant difference between the proportion of X (52.4%) and Y (47.6%) mature sperm in Ag(PMB)1 testes compared to the wild-type control (*p* = 0.255), therefore excluding the hypothesis that haploid germline cells carrying the X chromosome are removed following I-*Ppo*I cleavage. We also found that a small fraction of Ag(PMB)1 sperm showed both X and Y staining (3.6%) or no staining of X or Y (12.8%), likely due to chromosomal non-disjunction events during meiosis (Table 1 and Figure S1).

**Table 1.** Summary of proportions of sperm carrying X or Y chromosome, both X and Y chromosomes (X + Y) or neither X/Y chromosomes (∅) resulting from FISH analysis of testes and spermathecae post-copulation (left and middle column), and proportions of female and male progenies from Ag(PMB)1 and wild-type males crossed to wild-type G3 females (right column). Proportions of Y and X-bearing sperm and males and females are indicated as percentages and the total number of DAPI positive cells or adult mosquitoes counted are shown in parenthesis.

To determine whether X and Y-bearing sperm were successfully transferred in equal ratio to wild-type females, FISH was performed on postcopulatory sperm contained in the spermathecae (the female organ for sperm storage) 7–10 days after mating with Ag(PMB)1 males. Confocal analysis showed that a higher proportion of the sperm transferred to the spermathecae by Ag(PMB)1 males carried the Y chromosome (61.2%), though this bias was not significantly different from the wild-type control (60.2%; *p = *0.974), excluding the possibility of a differential transfer of sperm carrying the I-PpoI-shredded X chromosome. A small proportion of the sperm transferred by Ag(PMB1) males to wild-type spermathecae also carried non-disjunction signatures: 1.3% of cells showed both X and Y staining whilst 4.4% of DAPI-stained sperm showed no staining of either X or Y chromosomes. Despite no detection of non-disjunction signatures in the sperm contained in wild-type testes, probably due to the small sample size tested, 5.2% and 1.7% of the wild-type sperm transferred to spermathecae showed double-staining or no staining of X and Y chromosomes respectively, indicating that low frequencies of non-disjunctions normally occur during *A. gambiae* spermatogenesis. Our FISH analysis showed that more Y-bearing than X-bearing sperm were transferred to female spermathecae from both Ag(PMB)1 or wild type males. This may indicate that Y-bearing sperm is naturally less competitive compared to X-bearing sperm. Therefore, the preferential transfer of Y-bearing sperm to the spermathecae could be the result of a naturally occurring precopulatory mechanism evolved to guarantee mendelian inheritance of X:Y chromosomes from the male parent to maintain an unbiased sex ratio.

Considering that it was not possible to determine cellular viability of the Ag(PMB)1 X-bearing sperm counted in our confocal analysis, we also performed vigor analysis to test motility of sperm bundles in the spermathecae, which also showed no statistical difference between Ag(PMB)1 and the wild-type males (*p* = 0.063). However, sperm motility was analyzed from the entire sperm bundle within the spermatheca and not from individual sperm cells possibly diluting the resulting signal (Figure S2).

Previous studies showed that *Drosophila melanogaster* females mated with males heterozygous for the well-studied killer meiotic driver carry dysfunctional (*i.e*. dead) sperm [22]. Conversely, in the roundworm *Caenorhabditis elegans* apoptosis does not occur in response to spermatogenic meiotic defects due to the inability to activate the downstream cytotoxic caspase CED-3 [23]. The postcopulatory transfer of aneuploid sperm, detected from both *A. gambiae* Ag(PMB)1 and wild-type males, indicates that sperm does not have to be fully functional to be transferred to female mosquitoes after copulation. Also, in some *Drosophila* species, only long sperm fertilizes the eggs, despite males producing both long and short nucleated mature sperm [24,25], suggesting a possible role of the non-fertilizing mature sperm such as facilitating sperm transportation, nutritional provisions, or preventing receipt and storage of sperm from competing males [26]. Studies in *A. gambiae* showed that postcopulatory sperm competition may occur between polymorphic sperm of various lengths, where males with short sperm have higher reproductive success [27].

### Meiotic cleavage of both X and Y chromosomes in SD males does not reduce male fertility indicating post-copulation sperm competition as a mechanism regulating male-biased SD

**Figure 1.**
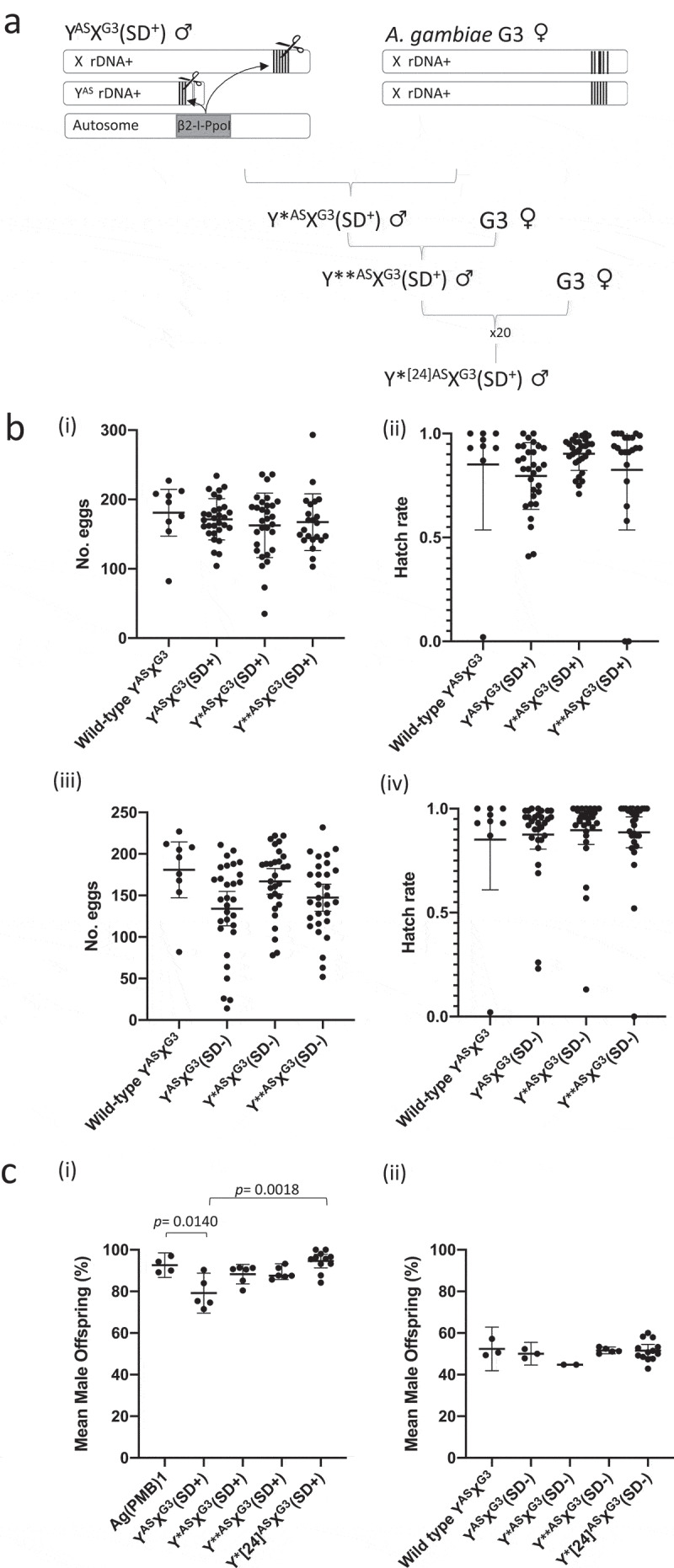
(A) Schematic representation of crosses to investigate the phenotypic effect of simultaneous meiotic cleavage of X and Y chromosome in *A. gambiae. A. gambiae* ASEMBO males were crossed to *A. gambiae* G3-derived Ag(PMB)1 females to generate Y^AS^X^G3^ (SD^+^) males carrying the autosomal insertion of the b2-tubulin::I-PpoI transgene and rDNA clusters on both sex chromosomes. The ASEMBO-derived rDNA cluster on the Y chromosome is expected to be smaller than the rDNA cluster in the X chromosome (Supplementary Figure 4). The I-PpoI nuclease is expected to cleave the 28S rDNA subunit present on both sex chromosomes during Y^AS^X^G3^(SD^+^) male meiosis. Consecutive crosses between Y^AS^X^G3^ (SD^+^) males and Ag(PMB)1 *A. gambiae* G3 females are expected to generate males carrying a Y chromosome previously exposed to I-PpoI cleavage (Y*^AS^, where ‘*’, ‘**’ ‘*^[24]^’ indicates I-*Ppo*I exposure for 1, 2 or 24 consecutive generations) and a nonexposed X chromosome (X^G3^) chromosome. (B) Dot plots summarising the number of eggs laid per female and the number of larvae hatching from SD+ males (i and ii), and non-transgenic sibling males (iii and iv), carrying I-PpoI nonexposed (Y*^AS^ or Y**^AS^) or exposed Y chromosomes (Y^AS^) crossed to wild type females.. Wild-type males were also crossed as a control in each set of crosses. (C) Dot plots showing the mean percentage of male progeny from Ag(PMB)1, Y^AS^X^G3^(SD^+^), Y*^AS^X^G3^(SD^+^), Y**^AS^X^G3^(SD^+^), and Y*[24]^AS^X^G3^(SD^+^) males. Mean values shown in A and B are provided in Supplementary Table 1. *P* values indicate significance between crosses according to one-way ANOVA analysis on ranks for hatch rates). Horizontal bars indicate average values and error bars show 95% confidence intervals

To further analyze the role of sperm competition in generating the SD male bias, males of the wild type *A. gambiae* ASEMBO strain were crossed to Ag(PMB)1 females to generate transgenic males, hereafter called Y^AS^X^G3^(SD^+^), carrying rDNA copies in both Y (Y^AS^) and X chromosomes (X^G3^) as well as the autosomal β2-tubulin::I-*Ppo*I transgene(SD). In Y^AS^X^G3^ (SD^+^) males, the β2-tubulin::I-*Ppo*I transgene is expected to simultaneously cleave the Y-linked and X-linked rDNA, thereby causing damage to both sex chromosomes (Figure 1(a)). Surprisingly, Y^AS^X^G3^ (SD^+^) males crossed to G3 wild-type females gave progeny without any significant reduction of fertility compared to wild-type ASEMBO males or non-transgenic siblings (Figure 1(b)). Yet only 79.2 % of the progeny of Y^AS^X^G3^ (SD^+^) males were found to be males, significantly lower than the 92.6 % male bias observed in the Ag(PMB)1 control strain, which only carries the rDNA target site on the X chromosome (*p* = 0.014). No significant difference in sex bias was observed in the progeny of Y^AS^X^G3^ (SD^−^) males, excluding the possibility of any potential mechanisms of genetic incompatibility that could interfere with the sex bias observed (Figure 1(c)).

To test the effect of repeated cleavage of Y-linked rDNA copies, Y^AS^X^G3^ (SD^+^) males were backcrossed to wild-type *A. gambiae* G3 females for up to 24 consecutive generations. Each generation, Y^AS^X^G3^ (SD^+^) males inherited an intact X^G3^ chromosome from the female wild-type parent and a Y^AS^ chromosome from the SD^+^ male parent, pre-exposed to I-PpoI-mediated meiotic cleavage (indicated as Y*^AS^, where ‘*’ specifies a previous exposure to I-PpoI cleavage in the male parent). After just one further generation of exposure to meiotic cleavage, the transgenic Y*^AS^X^G3^ (SD^+^) males generated a higher male bias (compared to Y^AS^X^G3^ (SD^+^) males) giving 88.3 % male progeny, not significantly different from the Ag(PMB)1 control where only the X chromosome is cleaved (*p* = 0.476). We further investigated this phenotype by measuring male bias from males carrying Y-linked rDNA pre-exposed to I-PpoI for a maximum of 24 consecutive generations together with a nonexposed X-linked rDNA. Fertility analysis showed no significant difference between the number of eggs laid (per mated female) or the number of larvae hatched from females mated to X^G3^ (SD^+^) males carrying a repaired Y^AS^ chromosome after one, two or 24 generations compared to the respective wild-type control (Figure 1(b) and Figure S3). Interestingly, SD^+^ males carrying Y^AS^ chromosomes exposed to I-PpoI for 24 consecutive generations (Y*^[24]AS^X^G3^ (SD^+^)), generated male ratios remarkably similar to the Ag(PMB)1 control (corresponding to 94.6% of males) and significantly higher than the 79.2% male bias observed in the progeny of Y^AS^X^G3^ (SD^+^) males (*p* = 0.002, Figure 1(c)). These results showed that the male bias gradually increased up to the values obtained when only the X chromosome is targeted. These findings suggest that the Y-linked rDNA may become gradually refractory to I-PpoI cleavage as a likely consequence of loss of entire rDNA units or accumulation of EJ repairs at the target site. Whilst both hypotheses are difficult to test due to the repetitiveness of the rDNA array and the presence of the X-linked unit in these males, we performed amplicon sequencing across the 28S I-PpoI recognition sequence in Y*^[24]AS^X^G3^ (SD^+^) males to determine whether the high male-bias may have been restored as a result of an accumulation of end joining (EJ) repairs at the Y-linked rDNA targets, which would be no longer recognized by the I-PpoI endonuclease. Despite the frequency of putative EJ events being diluted by the X chromosome-derived wild-type copies, two deletion events were found at frequencies 10^3^ times higher in 2 out of 5 Y*^[24]AS^X^G3^ (SD^−^) males tested compared to the control males carrying the nonexposed Y^AS^ chromosome; specifically, a 7 bp deletion (Del^121^), and a 22 bp deletion (Del^118^), that occurred within the I-PpoI cut site (Table S2). These two deletions were also found in the wild-type samples, at a 10^3^ lower frequency. This suggests that Del^121^,and Del^118^,may have been selected upon deletion of whole rDNA repeats, reducing the proportion of unmutated copies, or due to mechanism of molecular amplification of the EJ events, *e.g*. via homology directed repair (HDR) of cleaved targets using the resistant copy carrying the EJ mutation [28]. Repair of X chromosomes following meiotic I-PpoI cleavage has been previously detected in the surviving females generated by transgenic SD males with evident loss of rDNA copies. However, the remaining rDNA copies were still susceptible to cleavage after further exposure to I-*Ppo*I, indicating the presence of fully functional ribosomal units [9].

All together these results show that, in the described settings, I-PpoI-mediated meiotic cleavage of sex chromosomes does not affect *A. gambiae* male fertility and that the consequent male bias may likely result from reduced capacity of sperm carrying a damaged or misrepaired sex chromosome to fertilize the female oocyte.

## Conclusion

In this work we investigated the mechanisms regulating synthetic sex ratio distortion in the germline of fully fertile Ag(PMB)1 *A. gambiae* male-bias line. FISH staining of pre- and postcopulatory sperm revealed no difference between the number of X-bearing and Y-bearing sperm compared to the wild-type control, thereby suggesting the absence of germline checkpoints able to remove damaged sperm before reaching maturation. We also studied the impact of meiotic cleavage on the preferential inheritance of the sex chromosomes in transgenic males carrying rDNA clusters in both Y and X chromosomes. Consistent with the hypothesis that sperm carrying I-PpoI damaged sex chromosome are able to reach full maturation, these males showed normal fertility compared to wild type, indicating post-copulation sperm competition as the most plausible mechanism regulating synthetic sex-ratio distortion. Nevertheless, the significantly reduced male bias obtained from these males indicates that Y-sperm maintained a selective advantage over the X-sperm suggesting that the number of cleavable rDNA targets, expected to be lower in the Y chromosome compared to the X in these males, may have a direct effect on sperm competition as a phenotypic consequence of overall chromosomal damage. This hypothesis was also supported by the increased male bias obtained from SD^+^ males carrying a wild-type X chromosome and a susceptible Y^AS^ chromosome pre-exposed to I-PpoI activity for up to 24 generations. Our results also indicate that the presence of rDNA repeats in the Y chromosome does not affect sex distortion and fertility capacities of *A. gambiae* synthetic sex distorters highlighting the robustness of this technology for potential field applications.

## Methods

### Mosquito strains

Wild-type *A. gambiae* G3, *A. coluzzii* Mopti and *A. gambiae* ASEMBO strains (MRA-112, MRA-763 and MRA-186 respectively) obtained from the Biodefense and Emerging Infections Research Resources Repository (BEI) were used in this study.

The rDNA locus of the G3 strain is exclusively located on the X chromosome, whereas the rDNA in the ASEMBO strain is located in both the X chromosome and another locus containing fewer repeat units of a mixture of S- and M-type [8] rDNA is located in the Y chromosome in the ASEMBO strain (Figure S4). The presence of rDNA on the ASEMBO Y chromosome is thought to be due to a recent recombination event between the sex chromosomes because Y-linked rDNA is absent in other *A. gambiae* lab strains, such as the G3 strain, which was isolated years earlier from West Africa in 1975, whereas the ASEMBO strain (also referred to as Asembo Bay or AB) was originally isolated from East Africa (Kenya) in 1997. Both *A. gambiae* ASEMBO and G3 strains are considered hybrid stocks with mixed features derived from both *A. gambiae* s.s. and *Anopheles coluzzii*.

The fully fertile transgenic *A. gambiae* strain (^gfp^124L-2) carries the autosomal male-bias β2-tubulin::I-*Ppo*I transgenes and the 3xP3-DsRed fluorescent marker on chromosome 3R [9] in a G3 background, and is referred to herein the Ag(PMB)1 strain. Transgenic mosquitoes were analyzed on a Nikon inverted microscope at a wavelength of 563 nm to detect DsRed expression (Filter 630/30 nm emission, 595 nm dichroic).

Mosquitoes were reared under standard condition of 28oC, 70% relative humidity, with 12:12 light:dark cycle, with access to fish food as larvae and 5% (weight/volume) glucose solution as adults, and adult females were fed on cow blood for egg production, and eggs were collected in saline water (dH_2_O and 0.1% pure salt). Protocols and procedures were approved by the Imperial College Animal Ethics Committee in compliance with UK Home Office regulations.

### *Fluorescence* in situ *hybridization (FISH)*

#### Testes dissections

Testes were dissected from late male Ag(PMB1) pupae and the wild-type *A. coluzzii* Mopti control on microscopic slides in hypotonic solution (0.075 M KCl). Tissues were fixed in modified Carnoy’s solution, covered in 50% propionic acid, immediately covered with a 22 × 22 mm coverslip, dipped in liquid nitrogen and serially dehydrated in 70%, 80% and 100% ethanol after removal of coverslips as previously described [8]. Sperm in testes from 4 males and at least 3 fields of view were counted per genotype. Counts represent elongated and flagellated sperm as it was not possible to differentiate between mature spermatozoa and elongating/flagellated intermediates.

#### Spermathecae dissections for FISH analysis

For each strain (wild-type *A. gambiae* G3 and transgenic Ag(PMB)1), 50 two-day-old adult male mosquitoes were introduced to a separate cage with 50 wild-type *A. gambiae* G3 females and mating was allowed for 5–7 days before females were immobilized at −20oC for 5 minutes and stored on ice in a petri-dish for dissections. Single females were placed on a glass slide and in 1x PBS solution at room temperature. Once the spermatheca was dissected using fine needles, excess tissue was removed, the PBS was removed using a piece of paper towel, and a drop of Modified Sperm Nutrient solution (NaCl, 0.081 g MgCl2.6H2O, 0.019 g CaCl2.2H2O, 0.090 g D-glucose, 0.238 g HEPES in 100 mL autoclaved dH20; Liang *et al*., in preparation) prewarmed at 37oC was added and covered with a 22 × 22 mm coverslip. Spermathecae were ruptured by gently tapping the coverslip and mature sperm were checked at 10/20x magnification. When sperm was observed, excess fluid was removed from around the coverslip using Whatman filter paper and slides were dipped in liquid nitrogen until samples stopped bubbling. Coverslips were removed swiftly with a sharp razor blade and transferred to fresh 50% ethanol pre-chilled at −20°C and stored in the 50% ethanol overnight. Slides were serially dehydrated in room temperature 70%, 90% and 100% ethanol for 5 minutes each, dried and stored at −20°C ready for FISH straining. Sperm in 2 spermathecae from Ag(PMB)1 crosses and 1 spermatheca from *A. gambiae* G3 cross (and at least 3 fields of view per spermatheca) were counted.

#### Imaginal disc dissections for FISH analysis

Imaginal discs of 4th instar *A. gambiae* ASEMBO larvae were dissected for mitotic chromosome preparations as previously described [29].

#### Probe preparation and FISH

Probes to perform FISH were generated by incorporating fluorescent labels during a PCR reaction with primers for amplifying *Zanzibar* and 18S as previously described [8]. FISH was performed as previously described [29]. Chromosomes were counter-stained with DAPI (Life Technologies, Carlsbad, CA, USA), kept in the dark for at least 2 hours, and visualized on an Olympus BX61 fluorescent microscope (Olympus, Tokyo, Japan) using BioView software (BioView Inc., Billerica, MA, USA) at 1000x magnification and processed as previously described [8].

### Sperm activity assay

Wild-type *A. gambiae* G3 females were allowed to mate for up to seven days with either Ag(PMB)1 or wild-type males. Females were collected and immobilized using CO_2_, their spermathecae dissected in PBS buffer, and sperm activity was scored blindly every 10s for 60 seconds as described [30,31].

### In vivo *phenotype assays: Eggs per female, hatch rate and percent males*

Pooled cages containing 60 females of each genotype were crossed to 60 wild-type *A. gambiae* G3 males. Females were provided with a blood meal 5–7 days later. Blood fed females were transferred to individual cups with filter paper containing salinated water lined with Whatman filter paper. Eggs were sprayed with water to induce synchronous hatching, and larvae were counted. Eggs were allowed 10 days after spraying to hatch before being classified as unhatched. In some cases where only 1 larva from over 100 eggs hatched, spermathecae of the female parent was dissected and the presence of the sperm bundle was confirmed (and was not visibly different to the usual size of the sperm bundle). All L1 larvae that hatched in the first 3-days were pooled per cross and a sample of at least three trays (100 L1 larvae per tray) was prepared. Larvae were reared at the same density (1 larva per mL) and the total numbers of male: female pupae were counted to calculate the percentage of males.

### Amplicon sequencing

To determine whether there was a difference between the frequency of mutations in the I-*Ppo*I recognition sequence of Y^AS^ following 24 rounds of I-*Ppo*I endonuclease exposure, we performed amplicon sequencing across the 28S I-*Ppo*I recognition sequence in five non-transgenic males carrying Y^ASEMBO^ that were exposed to 24 rounds of I-*Ppo*I activity (Y*^[24]AS^X^G3^ (SD^−^); these were non-transgenic siblings of Y*^[24]AS^X^G3^ (SD^+^) males) and five wild-type *A. gambiae* G3-ASEMBO hybrids (carrying wildtype Y^AS^X^G3^).

GENEWIZ Amplicon-EZ deep sequencing was performed on individual insects: five F23 Y^ASEMBO^Ag(PMB)1 males and five wild-type Y^ASEMBO^X^G3^ males. DNA was extracted using the Wizard® Genomic DNA Purification Kit Protocol according to the manufacturers protocol except that the DNA was eluted in 20 μL DNAse/RNAse-free H_2_O. Primers RH99 and RH100 targeting 374 bp of rDNA sequence including the I-*Ppo*I 28S target site were designed to contain the llumina Nextera Transposase Adapters to tag the amplicon for subsequent library preparation and sequencing. rDNA locus-specific RH99 and RH100 primers showing underlined overhang Illumina Nextera Transposase Adapters:

TCGTCGGCAGCGTCAGATGTGTATAAGAGACAGCAAAGCATTGTGATGGCCC and GTCTCGTGGGCTCGGAGATGTGTATAAGAGACAGGGCAACAGTAAGAGTGGTGGT respectively. KAPA HiFi Hotstart Ready Mix PCR Kit (Kapa Biosystems) was used to amplify 20 ng genomic DNA per sample in 50 μL reactions. To maintain the proportion of reads corresponding to particular alleles at the target site, the PCR reaction was performed under non-saturating conditions for 20 cycles instead of 35 before 25 μL were removed and stored at −20oC for amplicon sequencing. The remaining 25 μL reactions were run for an additional 20 cycles and used to verify the amplification on a 1% agarose gel. Amplicon sequencing was performed by Polo d’Innovazione di Genomica, Genetica e Biologia (Italy). Data was analyzed using CRISPResso [32] focussing on 100bp region flanking the I-*Ppo*I target site (50 bp upstream and 50 bp downstream of the cut site).

### Statistics

The Pearson’s chi-squared (*χ*^2^) analysis was used to compare X:Y sperm counts [33]. Percentage male offspring and hatch rates were converted to arcsine square root values before Welch’s ANOVA was used for unequal variance as assessed by the Levene’s test and the Games-Howell *post-hoc* test was used to identify differences between groups using the IBM SPSS Statistics program (version 24.0; SPSS Inc., Chicago, USA). Prism 8 (version 9.2.0; GraphPad Software, Inc., San Diego, USA) was used for one-way ANOVA to assess the number of eggs or larvae per females, Kruskal-Wallis H test (one-way ANOVA on ranks) to assess the arcsine transformed hatch rate of eggs, and the Mann Whitney test was used to compare the arcsine transformed frequency of reads from amplicon sequencing of the 28S region targeted by the I-*Ppo*I endonuclease since arcsine-transformed frequency of reads for both groups did not meet the assumption of normality as assessed by the Shapiro-Wilk’s test.

## Supplementary Material

Supplemental Material

## References

[1] World malaria report 2018. Geneva: World Health Organization; 2018.

[2] Newton ME, Wood RJ, Southern DI. A cytogenetic analysis of meiotic drive in the mosquito, Aedes aegypti (L.). Genetica. 1976;46:297–318.

[3] Sweeny TL, Barr AR. Sex ratio distortion caused by meiotic drive in a mosquito, Culex pipiens L. Genetics. 1978;88:427–446. 17248804 10.1093/genetics/88.3.427 PMC1224591

[4] Hamilton WD. Extraordinary sex ratios. Science. 1967;156:477–488. 6021675 10.1126/science.156.3774.477

[5] Burt A. Site-specific selfish genes as tools for the control and genetic engineering of natural populations. Proc Biol Sci. 2003;270:921–928. 12803906 10.1098/rspb.2002.2319 PMC1691325

[6] Collins FH, Besansky NJ, Mendez MA, et al. A ribosomal RNA gene probe differentiates member species of the Anopheles gambiae complex. Am J Trop Med Hyg. 1987;37:37–41. 10.4269/ajtmh.1987.37.37 2886070

[7] Marchi A, Pili E. Ribosomal RNA genes in mosquitoes: localization by fluorescence in situ hybridization (FISH). Heredity (Edinb). 1994;72:599–605. 7914517 10.1038/hdy.1994.83

[8] Hall AB, Papathanos P-A, Sharma A, et al. Radical remodeling of the Y chromosome in a recent radiation of malaria mosquitoes. Proc Natl Acad Sci. 2016;113:201525164. 10.1073/pnas.1525164113 PMC4839409 27035980

[9] Galizi R, Doyle LA, Menichelli M, et al. A synthetic sex ratio distortion system for the control of the human malaria mosquito. Nat Commun. 2014;5:1–8. 10.1038/ncomms4977 PMC4057611 24915045

[10] Windbichler N, Papathanos PA, Crisanti A. Targeting the X chromosome during spermatogenesis induces Y chromosome transmission ratio distortion and early dominant embryo lethality in Anopheles gambiae. PLoS Genet. 2008;4:e1000291. 19057670 10.1371/journal.pgen.1000291 PMC2585807

[11] Galizi R, Hammond A, Kyrou K, et al. A CRISPR-Cas9 sex-ratio distortion system for genetic control. Sci Rep. 2016;6:31139. 27484623 10.1038/srep31139 PMC4971495

[12] Facchinelli L, North AR, Collins CM, et al. Large-cage assessment of a transgenic sex-ratio distortion strain on populations of an African malaria vector. Parasit Vectors. 2019;12:70. 30728060 10.1186/s13071-019-3289-y PMC6366042

[13] Huang Y, Magori K, Lloyd AL, et al. Introducing desirable transgenes into insect populations using Y-linked meiotic drive - a theoretical assessment. Evolution (NY). 2007;61:717–726. 10.1111/j.1558-5646.2007.00075.x 17439607

[14] Alcalay Y, Fuchs S, Galizi R, et al. The potential for a released autosomal X-shredder becoming a driving-Y chromosome and invasively suppressing wild populations of malaria mosquitoes. bioRxiv. 2019:860551. DOI:10.1101/860551 PMC8698249 34957064

[15] Taxiarchi C, Kranjc N, Kriezis A, et al. High-resolution transcriptional profiling of Anopheles gambiae spermatogenesis reveals mechanisms of sex chromosome regulation. Sci Rep. 2019;9. DOI:10.1038/s41598-019-51181-1 PMC6795909 31619757

[16] Cloutier JM, Turner JMA. Meiotic sex chromosome inactivation. Curr Biol. 2010;20:R962–3. 21093783 10.1016/j.cub.2010.09.041

[17] Gantz VM, Jasinskiene N, Tatarenkova O, et al. Highly efficient Cas9-mediated gene drive for population modification of the malaria vector mosquito *Anopheles stephensi*. Proc Natl Acad Sci. 2015;2015:21077. 10.1073/pnas.1521077112 PMC4679060 26598698

[18] Hammond A, Galizi R, Kyrou K, et al. A CRISPR-Cas9 gene drive system targeting female reproduction in the malaria mosquito vector *Anopheles gambiae*. Nat Biotechnol. 2016;34:78–83. 10.1038/nbt.3439 PMC4913862 26641531

[19] Burt A. Heritable strategies for controlling insect vectors of disease. Philos Trans R Soc Lond B Biol Sci. 2014;369:20130432. 24821918 10.1098/rstb.2013.0432 PMC4024225

[20] Kyrou K, Hammond AM, Galizi R, *et al*. A CRISPR–Cas9 gene drive targeting doublesex causes complete population suppression in caged Anopheles gambiae mosquitoes. Nat Biotechnol. 2018;36:1062–1066. 30247490 10.1038/nbt.4245 PMC6871539

[21] Simoni A, Hammond AM, Beaghton AK, et al. A male-biased sex-distorter gene drive for the human malaria vector Anopheles gambiae. Nat Biotechnol. 2020:1–7. DOI:10.1038/s41587-020-0508-1 32393821 PMC7473848

[22] Larracuente AM, Presgraves DC. The selfish segregation distorter gene complex of Drosophila melanogaster. Genetics. 2012;192:33–53. 22964836 10.1534/genetics.112.141390 PMC3430544

[23] Jaramillo-Lambert A, Harigaya Y, Vitt J, et al. Meiotic errors activate checkpoints that improve gamete quality without triggering apoptosis in male germ cells. Curr Biol. 2010;20:2078–2089. 20970339 10.1016/j.cub.2010.10.008 PMC3005853

[24] Snook RR, Markow TA, Karrt TL. Functional nonequivalence of sperm in Drosophila pseudoobscura. Proc Natl Acad Sci. 1994;91:11222–11226. 7972038 10.1073/pnas.91.23.11222 PMC45199

[25] Snook RR, Karr TL. Only long sperm are fertilization-competent in six sperm-heteromorphic Drosophila species. Curr Biol. 1998;8:291–294. 9501071 10.1016/s0960-9822(98)70112-5

[26] Swallow JG, Wilkinson GS. The long and short of sperm polymorphisms in insects. Biol Rev Camb Philos Soc. 2002;77:S1464793101005851. 10.1017/s1464793101005851 12056745

[27] Voordouw MJ, Koella JC, Hurd H. Intra-specific variation of sperm length in the malaria vector Anopheles gambiae: males with shorter sperm have higher reproductive success. Malar J. 2008;7:214. 18939985 10.1186/1475-2875-7-214 PMC2605757

[28] Seol J-H, Shim EY, Lee SE. Microhomology-mediated end joining: good, bad and ugly. Mutat Res/Fundam Mol Mech Mutagen. 2018;809:81–87. 10.1016/j.mrfmmm.2017.07.002 PMC6477918 28754468

[29] Timoshevskiy VA, Sharma A, Sharakhov IV, et al. Fluorescent in situ hybridization on mitotic chromosomes of mosquitoes. J Vis Exp. 2012:e4125. DOI:10.3791/4215 PMC3671840 23007640

[30] Ekechukwu NE, Baeshen R, Traorè SF, et al. Heterosis increases fertility, fecundity, and survival of laboratory-produced F1 hybrid males of the malaria mosquito *Anopheles coluzzii*. G3 (Bethesda). 2015;5:2693–2709. 26497140 10.1534/g3.115.021436 PMC4683642

[31] Ekechukwu NE, Tripet F. Current versus future reproductive investment adaptive responses in adult Anopheles coluzzii malaria mosquitoes: hydric-stressed males give it all. Parasit Vectors. 2019;12:377. 31358037 10.1186/s13071-019-3608-3 PMC6664720

[32] Pinello L, Canver MC, Hoban MD, et al. Analyzing CRISPR genome editing experiments with CRISPResso HHS public access author manuscript. Nat Biotechnol. 2016;34:695–697. 27404874 10.1038/nbt.3583 PMC5242601

[33] Pearson K. On the criterion that a given system of deviations from the probable in the case of correlated system of variables is such that it can be reasonably supposed to have arisen from random sampling. Philos Mag. 1900;50:157–175.

