# Supplementary material for: Cellular mechanisms regulating synthetic sex ratio distortion in the Anopheles gambiae germline
Source: Pathog Glob Health. 2020 Oct 12;114(7):370–8. doi: 10.1080/20477724.2020.1803628 (PMC7580827; doi:10.1080/20477724.2020.1803628)
Supplement: Supplemental Material [file YPGH_A_1803628_SM1512.docx]

## Supplementary Figure 1

Figure showing FISH of X-linked 18S rDNA (red) and the Y-linked retrotransposon *Zanzibar* (green) performed on chromosomes of sperm cells from Ag(PMB)1 testes and wild-type female spermathecae post-copulation with Ag(PMB)1 males. Chromosomes were counterstained with DAPI nuclear stain (blue); white arrow shows an example of sperm lacking both X and Y chromosomes. Scale bars, 2 μm in both images.

## Supplementary Figure 2

Figure showing the median vigour scores of sperm bundles observed in spermathecae from females post-copulation with Ag(PMB)1 or wild-type males ranked as 0 (no activity), 1 (very low), 2 (low), 3 (medium), and 4 (high). The females examined in this experiment were crossed to either Ag(PMB)1 or wild-type males (n=27 spermathecae per cross). Vigour scores were observed 6x per each sample replicate (every 10 seconds for a total of 1 min). Statistical significance between median sperm vigour values from Ag(PMB)1 (3.667) and wild-type (4) males was assessed using a Mann-Whitney test (*p*= 0.063). In the boxes, lower and upper edges represent the 25th and 75th percentiles and whiskers are equivalent to 25^th^-75^th^ quartile ±1.5* interquartile range.

## Supplementary Figure 3

Dot plots showing number of larvae per female from (i) transgenic and (ii) siblings lacking the β2-tubulin::I-PpoI allele. Horizontal bars indicate the mean values (summarised in **Supplementary Table 1**) and error bars indicate 95% confidence intervals. Significance was measured using one-way ANOVA analysis (*p*<0.05).

## Supplementary Figure 4

Figure showing FISH of X-linked 18S rDNA (red) and the Y-linked retrotransposon *Zanzibar* (green) performed on chromosomes of mitotic cells from imaginal disks (top) and sperm cells from testis (bottom) of *An. gambiae* ASEMBO. Chromosomes were counterstained with DAPI nuclear stain (blue); dashed white arrow shows the smaller rDNA cluster on the Y chromosome; white boxes show examples of Y-bearing sperm (rDNA^+^ and *Zanzibar*^+^) and X-bearing sperm (rDNA^+^). Scale bars, 2 μm in all images.

## Supplementary Table 1: Mean values from phenotypic analysis of each genotype tested

Table showing eggs per female, larvae per female, hatch rate and male-bias mean values per each genotype tested. The relative complete datasets are shown in **Figure 1B** and **Supplementary Figure 3**.

## Supplementary Table 2: Amplicon sequencing across the I-*Ppo*I target site

Table showing the results of amplicon sequencing. The frequency of the 11 most abundant non-reference reads (indels), indicated as a portion of the total reads per sample, are shown below for 5 Y*^[24]AS^X^G3^(SD^-^) males and the 5 wild-type Y^AS^X^G3^ males. The number following each deletion (DEL) or insertion (IN) indicates the position of the first nucleotide inserted or deleted within the 374 bp amplicon sequence. Highlighted in grey are the two indels within the I-*Ppo*I target site (DEL^121^ and DEL^118^) showing frequencies above the 0.00125 threshold applied (1/800 representing 700 X-rDNA repeats and 100 Y-rDNA repeats) are in grey, though these deletions were not detected in all samples.

|  | Y*^[24]AS^X^G3^males . | | | | | Wild-type Y^AS^ X^G3^males . | | | | |
| --- | --- | --- | --- | --- | --- | --- | --- | --- | --- | --- |
| Indels | 1 | 2 | 3 | 4 | 5 | 1 | 2 | 3 | 4 | 5 |
| Del^121^ |  | 0.001856 |  | 0.002038 |  |  |  | 0.000001 |  | 0.000002 |
| Del^118^ |  | 0.001585 |  |  |  |  | 0.000001 |  |  | 0.000001 |
| DEL^134-A^ | 0.000149 | 0.000128 | 0.000159 | 0.000147 | 0.000150 | 0.000158 | 0.000135 | 0.000292 | 0.000196 | 0.000147 |
| DEL^125-A^ | 0.000064 | 0.000068 | 0.000061 | 0.000068 | 0.000085 | 0.000074 | 0.000082 | 0.000066 | 0.000062 | 0.000059 |
| IN^134-A^ | 0.000007 | 0.000009 | 0.000002 | 0.000005 | 0.000008 | 0.000053 | 0.000005 | 0.000004 | 0.000005 | 0.000001 |
| DEL^123-T^ | 0.000041 | 0.000048 | 0.000028 | 0.000048 | 0.000049 | 0.000049 | 0.000041 | 0.000039 | 0.000036 | 0.000050 |
| DEL^127-G^ | 0.000032 | 0.000005 | 0.000002 | 0.000003 | 0.000004 | 0.000002 | 0.000005 | 0.000002 | 0.000005 | 0.000002 |
| IN^117+A^ | 0.000013 | 0.000009 | 0.000023 | 0.000010 | 0.000004 | 0.000005 | 0.000011 | 0.000015 | 0.000011 | 0.000008 |
| DEL^122-^  ^CTTAAGG^ | Not detected | 0.000020 | Not detected | 0.000017 | Not detected | Not detected | Not detected | Not detected | Not detected | Not detected |
| DEL^122-^ ^CTTAAGGT^  ^AGCCAAAT^  ^GCCTCG^ | Not detected | 0.000017 | Not detected | Not detected | Not detected | Not detected | Not detected | Not detected | Not detected | Not detected |
| DEL^132^ | Not detected | 0.001856 | Not detected | 0.002038 | Not detected | Not detected | Not detected | 0.000001 | Not detected | 0.000002 |
| IN^120+A^ | 0.000004 | 0.000006 | 0.000016 | 0.000007 | 0.000004 | 0.000008 | 0.000005 | 0.000004 | 0.000006 | 0.000002 |
